# Unique case report of a chromomycosis and *Listeria* in soft tissue and cerebellar abscesses after kidney transplantation

**DOI:** 10.1186/s12879-017-2386-9

**Published:** 2017-04-20

**Authors:** J. Tourret, N. Benabdellah, S. Drouin, F. Charlotte, J. Rottembourg, N. Arzouk, A. Fekkar, B. Barrou

**Affiliations:** 1 Département d’Urologie, Néphrologie et Transplantation, AP-HP, Groupe Hospitalier Pitié-Salpêtrière Charles Foix. 47-83, Bd de l’Hôpital, 75013 Paris, France; 2 Service d’Anatomie Cytologie Pathologique, Paris, France; 3 Service de Parasitologie Mycologie, Paris, France; 4 Sorbonne Universités, UPMC Univ Paris 06, Paris, France; 5 Sorbonne Paris Cité, Univ Paris Nord, IAME, INSERM UMR 1137, Paris, France; 6 INSERM U1135, CNRS ERL 8255, Centre d’Immunologie et des Maladies Infectieuses (CIMI-Paris), Paris, France

**Keywords:** Case report, Kidney transplantation, Tropical pathology, *Fonsecaea*, *Listeria*, Antifungal therapy

## Abstract

**Background:**

Chromomycosis is a rare mycotic infection encountered in tropical and subtropical regions. The disease presents as a slowly-evolving nodule that can become infected with bacteria. Here, we describe a unique association of abscesses caused by a chromomycosis and *Listeria monocytogenes* in a kidney transplant recipient, and didactically expose how the appropriate diagnosis was reached.

**Case presentation:**

A 49-year old male originating from the Caribbean presented a scalp lesion which was surgically removed in his hometown where it was misdiagnosed as a sporotrichosis on histology, 3 years after he received a kidney transplant. He received no additional treatment and the scalp lesion healed. One year later, an abscess of each thigh due to both *F. pedrosoi* and *L. monocytogenes* was diagnosed in our institution. A contemporary asymptomatic cerebellar abscess was also found by systematic MRI. An association of amoxicillin and posaconazole allowed a complete cure of the patient without recurring to surgery. Histological slides from the scalp lesion were re-examined in our institution and we retrospectively concluded to a first localisation of the chromomycosis. We discuss the possible pathophysiology of this very unusual association.

**Conclusion:**

In this case of disseminated listeriosis and chromomycosis, complete cure of the patients could be reached with oral anti-infectious treatment only.

## Background

Chromoblastomycosis is a chronic fungal disease mostly localised to the skin and the subcutaneous tissue. It is caused by saprophytic black moulds (also called dematiaceous fungi) which are characterised by the presence of a melanin pigment in the cell wall [1]. The disease is more frequent in humid tropical and subtropical regions, often in rural areas where the fungi are found in soils and plant debris [2, 3]. The fungi which is the most frequently responsible for infections in human is *Fonsecaea pedrosoi*, but others such as *Cladophialaphora carrionii*, *Phialophora verrucosa*, *Rhinocladiella aquaspersa*, *F. compacta*, *Exophiala dermatitidis*, *E. jeanselmei* and *E. spinifera*, have also been identified as pathogens for a long time, both in immunocompetent and immunocompromised hosts, such as transplant recipients [4–8].

The infection usually develops after a traumatic inoculation of the skin, the most exposed areas being the lower limbs. Farmers and people of low socioeconomic status are more likely to develop the disease [9]. After local inoculation, the infection presents as a small papule that slowly enlarges into verrucous nodules. The lesions are generally not painful. Nodules can progress to cauliflower-like masses, and can ulcerate. Complications include secondary bacterial infection and chronic lymphedema. The fungal infection itself can spread by lymphatic or hematogenous dissemination, producing distant metastatic lesions [10]. If not promptly diagnosed, chromoblastomycosis evolves into a chronic infection which may have several complications, such as difficulties in the therapeutic management due to extensive lesions [11]. There is a possible association with epidermoid carcinoma of the affected skin areas [12].

## Case presentation

A 49-year old male patient received a deceased donor renal allograft in our institution. He was born and raised in Haiti, immigrated in France 2 years before the kidney transplantation, but maintained regular trips to Haiti. He had a history of long-standing hypertension. His chronic kidney disease was discovered in France, when it had already reached end stage. Kidney transplantation induction therapy consisted in rabbit anti-thymocyte globulines, and one steroid pulse of 1000 mg. Maintenance immunosuppression comprised tacrolimus, mycophenolate mofetil and prednisone (20 mg daily for the first 3 months, progressively tapered to 5 mg per day at month 9 post-transplantation). The lowest serum creatinine was of 140 μmol/l. Five months after transplantation, the patient acquired diabetes mellitus which required insulin therapy.

Five years after the transplantation, while travelling to his hometown in Haiti, a tumour developed in a couple of weeks on the scalp. The tumour was surgically removed, and the histological analysis indicated a sporotrichosis. We were unable to retrieve any data about potential microbiologic culture or molecular biology analysis of the scalp lesion. The patient only brought back the histological slides that were processed in Haiti to our institution. No complementary treatment was administered and the lesion healed completely.

One year later, the patient was admitted to our institution after a 1-month stay in Haiti, because of fever and one cutaneous lesion on each thigh. There was no history of local trauma. Clinical examination revealed an erythematous area of 10 × 5 cm on each thigh. There was no crural lymphadenopathy.

Renal function was stable with a serum creatinine of 165 μmol/l. Plasma C-reactive protein was at 59 mg/L. There was neither anaemia nor leucocytosis. Diabetes was decompensated with an HbA1c at 10.5%. The day after admission, blood cultures yielded *Listeria monocytogenes*.

**Fig. 1:**
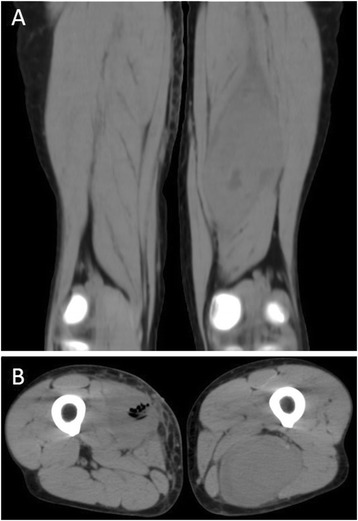
CT scan views of the thigh abscesses. The infection presents as an isodense image located within the muscles with a small necrotic centre. **a**: coronal view of the left thigh abscess. **b**: axial view of the left and right abscesses. The presence of air in the right thigh abscess is due to a wick that was left to ensure drainage of the abscess

Soft tissue ultrasound and computed tomography showed an abscess of the right vast medial muscle measuring 120 × 45 × 40 mm, and of the left semi-membranous muscle measuring 150 × 70 × 55 mm (Fig. 1).

**Fig. 2:**
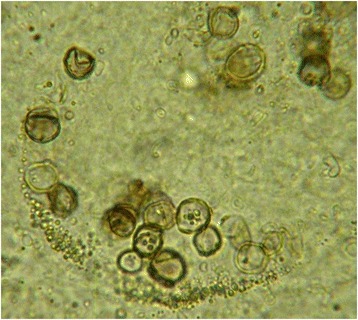
Direct examination of the pus drawn from the right thigh abscess. The microscopic examination showed fungal element called fumagoid cells (also called muriform cells or sclerotic bodies), which are indicative of chromoblastomycosis. Fumagoid cells are rounded brown pigmented septated structures with internal inclusions (optical microscopy, 40 X magnification)

**Fig. 3:**
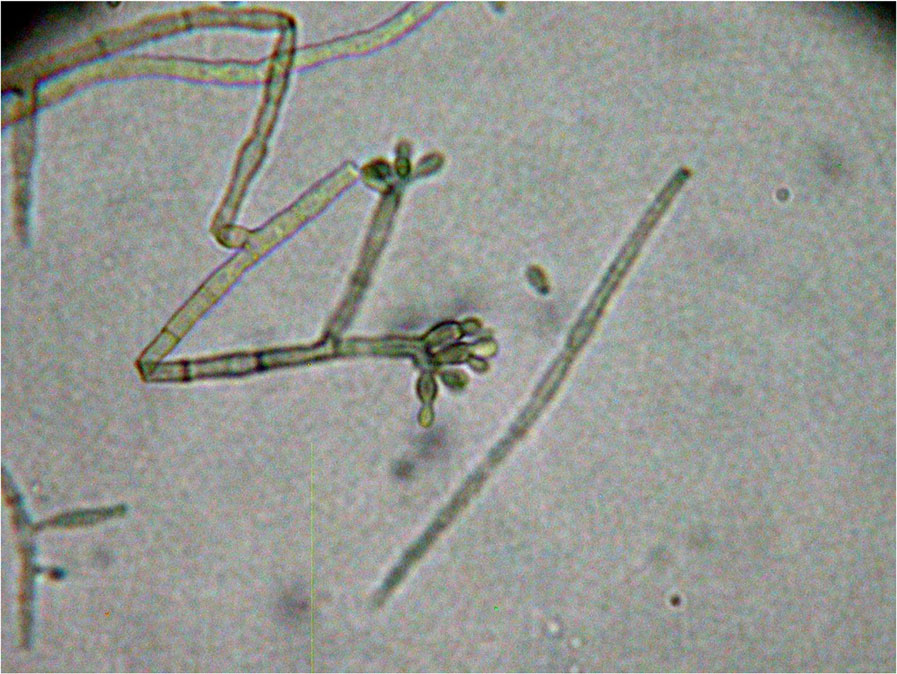
Microscopic examination of the fungal culture collected from the right thigh lesion. Conidiophore indicative of *Fonsecae sp.* are visible (optical microscopy, 40 X magnification)

Puncture of the abscess of the right thigh recovered a serous hematic and cloudy liquid. Direct microscopic examination of this pus evidenced sclerotic body or fumagoid cells (also called Medlar bodies) that are pathognomonic for chromoblastomycosis [13] (Fig. 2). Pus culture raised both *L. monocytogenes* and black mould colonies that were identified as *Fonsecae sp.* (Fig. 3). ITS sequencing (using the following primers: forward: 5′- TCCGTAGGTGAACCTGCGG- 3′: reverse 5′-TCCTCCGCTTATTGATATGC-3′ and the Genbank database) revealed *F. pedrosoi*. Minimal inhibitory concentrations of antifungal drugs could not be determined because of the impossibility to grow the fungus.

Retrospective re-evaluation in our institution of the histological slides of the scalp lesion removed in Haiti eventually disclosed an aspect of chromomycosis.

**Fig. 4:**
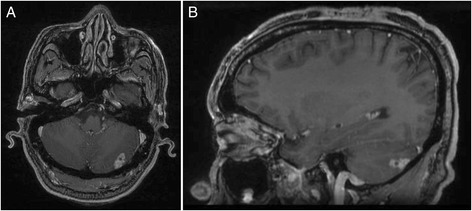
MRI of the central nervous system at diagnosis. Gadolinium enhanced T1 axial (**a**), and coronal (**b**) views. The images show an isolated left posterior cerebellar lesion consistent with an abscess

Chest and abdominopelvic computed tomography did not show any other infectious localization. In contrast, while the patient showed no neurologic sign, systematic cerebral magnetic resonance revealed a lesion of the peripheral left cerebellum, which was enhanced after gadolinium injection and which contained a necrotic centre (Fig. 4). Lumbar puncture was not performed.

**Fig. 5:**
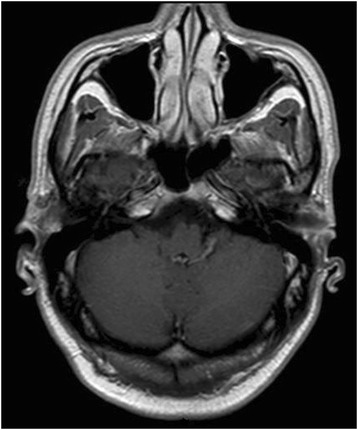
MRI of the central nervous system 3 months after amoxicillin treatment and posaconazole initiation. The imaging shows total regression of the cerebellar lesion

Treatment included percutaneous drainage of the abscess of the right thigh, intravenous followed by oral amoxicillin, and oral posaconazole (suspension; 800 mg/day). The abscess of the left thigh was not drained. The tacrolimus dose was decreased by 2/3, because of posaconazole-induced inhibition of cytochrome P450 3A4. Fever cessed a couple of days after anti-infectious treatment initiation, probably due the resolution of listeriosis with amoxicillin which was maintained for a total duration of 3 weeks. After 3 months of treatment with posaconazole, the abscess of the left thigh had significantly decreased, measuring only 5 × 3 cm, and the lesion of the right thigh seemed heeled. Brain MRI also showed a dramatic improvement after 3 months of treatment (Fig. 5). Posaconazole was discontinued after 12 months. Brain MRI showed no cerebellum sequela. Four years after the episode, the patient was asymptomatic with no recurrence of chromomycosis. Serum creatinine was stable at 136 μmol/l.

## Discussion

This is the first case that describes the association of a chromomycosis and a subcutaneous and haematogenous listeriosis.

As a black fungus, *F. pedrosoi* forms melanin, which is deposited in the cell wall and in the cytoplasmic structures [14]. Melanin plays a role in fungal pathogenicity, as it protects the cell from destruction by phagocytes and provides resistance to microbial lysis [15].

Chromomycosis management usually combines medical and surgical treatments. Medical therapy consists in a long course treatment with antifungal agents. The most frequently used agents are members of the azole group, i.e. itraconazole, voriconazole and posaconazole [16]. Another treatment option is terbinafine, which accumulates in the cutaneous tissue and showed a synergistic activity with itraconazole [17].

Thermotherapy or cryotherapy are effective for locally advanced disease [16]. The fungus is hard to eradicate, and treatment duration has been described up to 126 months [18]. Surgical treatment by local excision or debridement is indicated for extensive disease.

Immunosuppressive drugs such as steroids, mycophenolate mofetil and tacrolimus severely impair all components of immunity, including innate and adaptative immunity. Sousa Mda et al. showed that persistent infection by *F. pedrosoi* in mice was related to the deficiency of innate recognition of the fungus by Toll-like receptors (TLR) [19]. Administration of TLR ligands facilitated fungal clearance [19]. More recently, Wevers et al. showed that *F. monophora* could escape C-type lectin receptors on human dendritic cells [20]. We have shown that steroids were able to suppress C-type lectin in mice [21]. Finally, we also recently showed that calcineurin inhibitors might impair neutrophils-driven antifungal immunity [22]. Therefore, C-type lectin suppression, TLR inhibition, and neutrophil function deficiency are part of the putative mechanisms which could favour the development of chromomycosis in drug-induced immunosuppressed patients.

Because immunosuppressive drugs directly interact with T cell activation, an altered cellular immunity may also theoretically have participated in the development of chromomycosis in transplanted recipients. Because of its facultative intracellular life, *L. monocytogenes* is also favoured by cellular immunity deficiency [23]. The role of the humoral anti-fungal response is unknown, although high titters of anti-fungal antibodies have been reported in the course of severe fungal disease, followed by a decrease after effective treatment [24].

*Listeria monocytogenes* is a small Gram positive facultative anaerobic bacterium. It is found ubiquitously in soils, waters, and plants. It is very resistant to disinfection and is a food-borne pathogen. The bacterium can survive in refrigerators, is resistant to congelation and requires heat to be killed, which favours its environmental diffusion. The Centre for Disease Control in the United States estimates that *Listeria* is responsible for 1600 episodes of infection and 260 deaths annually [25]. The disease is more frequent in pregnant women, new-borns, older adults and immunocompromised hosts, such as cancer patients, people living with HIV/AIDS, dialysis patients, transplant recipients, and alcoholic and diabetic patients. In immunosuppressed hosts, listeriosis frequently presents as a septicaemia with meningitis [26]. Brain abscesses have also been described in transplant recipients [27, 28].

Cutaneous infections due to *Listeria monocytogenes* are rare (1), and usually due to direct inoculation from infected animals in high risk populations, such as farmers and veterinarians. In a recent review of 24 cases of cutaneous listeriosis, the most frequent risk factor was exposure to an aborted bovine foetus [29].

Extra-cutaneous lesions due to chromomycosis are very rare, but a few cases of cerebral abscesses, perhaps favoured by immunosuppression, have been reported [4, 30–32]. In our case, we can only speculate about the aetiology of the cerebellar lesion, as both listeriosis and chromomycosis can localize to the central nervous system. No puncture of the cerebellar abscess was performed because the patient was totally asymptomatic and because the evolution was rapidly favourable after treatment initiation. The risks of the puncture clearly outweighed the potential benefits. Even though cerebral involvement in listeriosis is much more frequent than in chromomycosis, the radiologic aspect observed in this case seemed to be slightly more compatible with a fungal lesion than with cerebral listeriosis, as the latter usually presents as multiple supratentorial microabscesses. A regional dissemination from the initial scalp fungal lesion to the cerebellum seems rather improbable because of spatial separation (the cutaneous lesion was localized at the top of the vertex) and of bone interposition with the absence of bone infection on brain MRI.

As both *Listeria* and chromomycosis muscle abscesses are also very rare, it is hard to determine whether primitive fungal abscesses were secondarily inoculated with *Listeria* during the septicemic phase, or if the pathophysiology worked the other way around.

Our case very didactically exposes the complexity of appropriate management of soft tissue abscesses in immunocompromised host. An isolated bacteriological examination of the pus would only have raised *Listeria*, and the anti-fungal treatment would not have been initiated. Therefore, in case of immunosuppression, it is very important to obtain bacterial, fungal and histological examination of collected samples. Similarly, without a systematic imaging of the brain, the cerebellar localisation would have been missed. As anti-fungal treatment duration is not well defined, sequential brain MRIs were part of the decision to stop treatment after one year.

## Conclusion

Cutaneous and central nervous system infections in transplant recipients can be due to a wide range of pathogens. Standard microbiology examination and cultures, histological analysis, imaging techniques, and molecular biology tools can be combined to reach an appropriate diagnosis. No recommendation exists for the treatment of invasive chromomycosis, but prolonged triazole treatment in selected cases can be sufficient.

## Abbreviations

HbA1c: Glycated haemoglobin
HIV/AIDS: Human immunodeficiency virus/acquired immune deficiency syndrome
MRI: Magnetic resonance Imaging
